# Data Resource Profile: Understanding the patterns and determinants of health in South Asians—the South Asia Biobank

**DOI:** 10.1093/ije/dyab029

**Published:** 2021-06-18

**Authors:** Peige Song, Ananya Gupta, Ian Y Goon, Mehedi Hasan, Sara Mahmood, Rajendra Pradeepa, Samreen Siddiqui, Gary S Frost, Dian Kusuma, Marisa Miraldo, Franco Sassi, Nick J Wareham, Sajjad Ahmed, Ranjit M. Anjana, Soren Brage, Nita G Forouhi, Sujeet Jha, Anuradhani Kasturiratne, Prasad Katulanda, Khadija I Khawaja, Marie Loh, Malay K Mridha, Ananda R Wickremasinghe, Jaspal S Kooner, John C Chambers, South Asia Biobank. Remaining authors are listed at the end of the article, Polly Page, Wnurinham Silva, Garudam R Aarthi, Saira Afzal, Sophie E Day, Bridget A Holmes, Rajan Kamalesh, Elisa Pineda, Fred Hersch, Baldeesh K Rai, Malabika Sarker, Jonathan Valabhji

**Affiliations:** 1 Department of Epidemiology and Biostatistics, School of Public Health, Imperial College London, London, UK; 2 School of Public Health, Zhejiang University School of Medicine, Hangzhou, China; 3 Institute of Endocrinology, Diabetes & Metabolism, Max Super Speciality Hospital (Devki Devi Foundation), New Delhi, India; 4 Centre for Non-communicable Diseases and Nutrition (CNCDN), BRAC James P Grant of Public Health, BRAC University, Dhaka, Bangladesh; 5 Department of Endocrinology & Metabolism, Services Institute of Medical Sciences, Services Hospital, Lahore, Pakistan; 6 Madras Diabetes Research Foundation, Chennai, India; 7 Faculty of Medicine, Imperial College London, London, UK; 8 Centre for Health Economics and Policy Innovation, Imperial College Business School, Imperial College London, London, UK; 9 Department of Economics and Public Policy, Imperial College Business School, Imperial College London, London, UK; 10 MRC Epidemiology Unit, Institute of Metabolic Science, University of Cambridge, Cambridge, UK; 11 Punjab Institute of Cardiology, Punjab, Pakistan; 12 Department of Public Health, Faculty of Medicine, University of Kelaniya, Ragama, Sri Lanka; 13 Department of Clinical Medicine, Faculty of Medicine, University of Colombo, Colombo, Sri Lanka; 14 Lee Kong Chian School of Medicine, Nanyang Technological University, Singapore, Singapore; 15 Ealing Hospital, London Northwest University Healthcare NHS Trust, London, UK; 16 National Heart and Lung Institute, Imperial College London, London, UK

## Data resource basics

Type 2 diabetes mellitus (T2DM) and cardiovascular disease (CVD) are leading and closely interlinked global health challenges.^1^ The burdens of T2DM and CVD are especially high in South Asia, one of the most populous and the most densely populated regions of the world.^2^^,^^3^ The prevalence of diabetes in South Asia has risen more rapidly than in other large geographical regions,^4^ and it is projected that South Asia will account for 40% of the global CVD burden by 2020.^5^ In addition, T2DM and CVD develop at an earlier age in South Asians than in their European counterparts^6^^,^^7^.

Identification of the primary risk factors for T2DM and CVD is central to the development of effective approaches for the prevention and treatment of chronic diseases such as T2DM and CVD.^8^ However, epidemiological data are currently sparse for South Asia, with evidence on the drivers of T2DM and CVD being predominantly based on cross-sectional studies that recorded a narrow range of exposures and without longitudinal assessments.^3^^,^^5^^,^^6^^,^^9^ The few available prospective studies are largely derived from investigations of South Asians residing in Western countries, and are further limited by small sample size and incomplete phenotypic characterization.^3^ To better understand the wide range of exposures that contribute to the development of T2DM and CVD in South Asians, a large-scale population-based study that collects information on demographic, lifestyle, clinical, environmental and genomic variables is needed.

To address this important need, we have established a unique cross-sectional population-based study focused on the South Asian population: the South Asia Biobank (SAB). SAB was launched in 2018 as a partnership between collaborating centres in Bangladesh, India, Pakistan, Sri Lanka and the UK.^10^ SAB includes rich demographic, lifestyle, clinical, environmental and phenotypic data and biological samples from more than 50 000 South Asian participants. This resource will enable a broad range of epidemiological research, including the development of prevention and treatment approaches, discovery of novel molecular biomarkers, risk stratification algorithms and innovative therapeutic approaches for better prevention of T2DM and CVD in South Asians. The specific initial objectives of SAB are as follows.

- Establish a network of non-communicable disease (NCD) surveillance sites in Bangladesh, India, Pakistan and Sri Lanka, using common protocols and platforms, in partnership with regional centres of excellence in South Asia.
- Complete structured health assessments on a representative sample of at least 50 000 South Asians aged 18 years and above, residing at all surveillance sites.
- Use the data to identify the genetic and environmental factors underlying non-communicable diseases in South Asians, and translate the findings into new approaches for maintenance of health and well-being.

The South Asia Biobank is conducted in accordance with the recommendations for physicians involved in research on human subjects, adopted by the 18th World Medical Assembly, Helsinki, 1964, and later revisions. Research approval was obtained from the Imperial College London Research Ethics Committee (reference: 18IC4698) and local institutional review boards in each of the participating countries.

## Data collected

**Figure 1.**
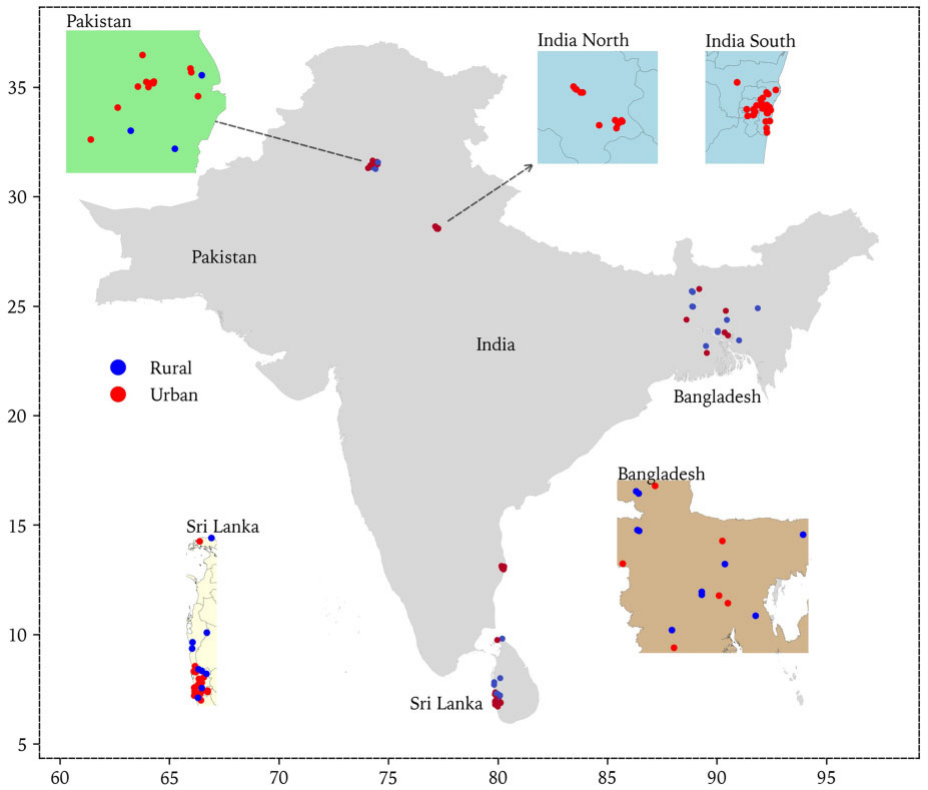
Locations of South Asia Biobank (SAB) surveillance sites

SAB is a cross-sectional population-based study that recruited participants from 118 surveillance sites that were centred on local primary community health care units in five study regions: Bangladesh, South India, North India, Pakistan and Sri Lanka. The locations of all surveillance sites are demonstrated in Figure 1. Recruitment started in November 2018 and ended in March 2020 (due to the pandemic of COVID-19).

We recruited men and women of self-reported South Asian ethnicity and aged 18 years and above. We excluded women who were currently pregnant, as well as people who were not permanent residents of the surveillance site (residence for 12 months or more required). We also excluded people with serious illness expected to reduce life expectancy to less than 12 months, those who planned to leave the surveillance site within the next 12 months and those unable or unwilling to give informed consent.

The surveillance sites at which recruitment occurred in each study region are summarized in Supplementary Table S1 (available as Supplementary data at *IJE* online). Governmental census data and available household listings were used, together with house-to-house visits by research teams and local primary care workers, to identify (enumerate) the resident population. All individuals in each household who met the inclusion criteria were invited to take part, and their demographic details were obtained in their households. We worked closely with senior community members (e.g. teachers, employers, religious leaders) to support and facilitate engagement in the study. Explanations of the project’s purpose were provided in writing and using videos, in relevant South Asian languages, supported by bilingual translators.

By March 2020 we recruited a total of 52 853 subjects: 13 954 from Bangladesh, 8620 from South India, 9469 from North India, 5875 from Pakistan and 14 935 from Sri Lanka. The response rate based on enumerated population in each surveillance site ranged from 17.6% in North India to 72.3 % in Pakistan (see Supplementary Table S2 for more details, available as Supplementary data at *IJE* online). The demographic structure of the study participants and the comparison with the National Population data in 2015, obtained from the United Nations Population Division, are shown in Table 1.^11^

**Table 1:** Characteristics of participants in South Asia Biobank (SAB), compared with reported national population distribution (United Nations Population Division)

Demographic characteristic	Bangladesh			India			Pakistan			Sri Lanka		
	National	SAB (*n* = 13 954)	*P*-value	National	SAB (*n *= 18 089)	*P*-value	National	SAB (*n* = 5875)	*P*-value	National	SAB (*n* = 14935)	*P*-value
Age			<0.001			<0.001			<0.001			<0.001
<30 years	30.28	15.71		28.53	13.07		34.32	15.74		21.17	10.56	
30-50 years	45.24	50.61		42.93	47.95		41.66	51.56		40.89	39.17	
50-70 years	19.08	29.25		23.09	33.37		19.04	30.88		29.98	40.74	
≥70 years	5.40	4.44		5.45	5.62		4.98	1.82		7.96	9.53	
Sex			<0.001			<0.001			<0.001			<0.001
Male	50.45	55.43		51.57	61.53		51.11	67.09		47.18	69.07	
Female	49.55	44.57		48.43	38.47		48.89	32.91		52.82	30.93	

Data are in percentages. The age category of ‘<30 years’ refers to ‘20–29 years’ in the United Nations Population Division and ‘18–29 years’ in SAB; chi- square goodness of fit testing was used to determine whether the age and sex proportions in SAB were similar to those in the general population.

### Measures

Participants were invited to attend the survey sites between 7 am and 11 am in the fasting state (water only after midnight). Structured assessments of participants were conducted in six complementary domains: (i) Registration and consent; (ii) Health and lifestyle questionnaire; (iii). Physical measurements; (iv) Biological samples (blood and spot urine); (v) Physical activity monitoring; and (vi) 24-h dietary recall. Procedures and training were standardized between countries and surveillance sites.

#### Registration and consent

Written, informed consent was obtained from all participants for data collection and inclusion in the research. Informed consent included permission for the data and samples collected to be used for chronic disease research, including data sharing with national and international bodies concerned with prevention and control of T2DM and CVD and for molecular epidemiological research. Consent was facilitated using videos (available in major South Asian languages). A unique study identity number (ID) was allocated to each participant.

#### Questionnaire

An interviewer-administered health and lifestyle questionnaire was used to collect information on behavioural risk factors (smoking, alcohol use, physical activity and consumption of fruits/vegetables), personal and family medical history, medications and socioeconomic status. The questionnaire was founded on the extended World Health Organization (WHO) STEPwise approach to surveillance questionnaire that is widely used in global disease surveillance and which was adapted for use in the South Asia context, through incorporating additional questions.^12^

#### Physical measurements

These included: (i) anthropometry (height, weight, waist and hip circumference and bio-impedance for body fat composition); (ii) blood pressure by digital device; (iii) cardiac evaluation by 12-lead electrocardiogram to identify arrhythmia, left ventricular hypertrophy and previous myocardial infarction; (iv) retinal photography for assessment of retinal disease, including hypertensive and diabetic retinopathy; and (v) respiratory evaluation by spirometry to assess for smoking/environment-related lung injury.

#### Biological samples

Using venesection by trained phlebotomists, 25 ml venous blood was collected and then distributed into ethylenediaminetetraacetic acid (EDTA), serum and citrate vacutainer tubes, and into tubes designed for RNA preservation (Tempus tube). Fasting glucose and cholesterol were measured by point of care tests. An oral glucose tolerance test was carried out in a subset of participants, enabling validation of diabetes classification. A spot urine sample (6 ml in three aliquots) was also collected for analysis of albuminuria and other biomarkers. Aliquots of whole blood, buffy coat, serum, EDTA plasma, citrate plasma and urine (Supplementary Table S3, available as Supplementary data at *IJE* online) were stored at −80°C for future molecular epidemiological research (including genomics), to investigate the mechanisms underpinning the development of T2DM, CVD and other complex diseases that are of importance to South Asians (including but not limited to: obesity, cancer, dementia, chronic obstructive pulmonary disease, chronic kidney disease).

#### Physical activity

This was also objectively quantified in 100-Hz resolution using a wrist-worn triaxial accelerometer, worn on participants’ non-dominant wrist for 7 days. This device is small, light-weight, wrist-watch-shaped, battery-powered and uses triaxial acceleration in gravitational units to infer participant movement. It has been used recently to measure physical activity patterns amongst 100 000 people in the UK Biobank study.^13^

#### Dietary intake

Consumption was recorded by interviewer-administered computerized 24-h dietary recall based on the multiple pass method using the Intake24 system [https://intake24.org/]. The system was specifically adapted for the South Asian context through incorporating extensive additional foods, drinks and dishes, and portion-size photographs relevant to the study settings. Adaptation was informed by research nutritionists and dieticians from each study centre and by the results of previous dietary surveys in the study locations. The implementation of this tool could enable the description of food and nutrient intakes, evaluation of intakes in comparison with guidelines and investigation of the link between diet and health endpoints.

All study participants received a report summarizing the clinically relevant results of their health assessment, together with an explanatory booklet and a link to access an explanatory video. Participants identified with significant health conditions (e.g. T2DM, hypertension) had the opportunity to discuss the results with the study team, and to be referred to an appropriate health care facility for further assessment, counselling or treatment.

### Environmental mapping

In each surveillance site, an environmental mapping exercise was carried out. The aim was to characterize the built environment in terms of retailers and advertisements for food and tobacco and physical activity facilities. The methodologies were adapted from food modules conducted by: the International Network for Food and Obesity/NCDs Research, Monitoring and Action Support; the Maryland Food Systems Map conducted by the Johns Hopkins Center for a Livable Future; and the World Health Organization Framework Convention on Tobacco Control.^14–16^ In addition to geolocations, the main variables included food (e.g. fruit, vegetables, confectionery), drinks (e.g. soft drinks, sugar-free drinks) and tobacco products (e.g. cigarette, beedi) being sold or advertised. Data collection used KoboToolBox for Android [https://www.kobotoolbox.org] and covered each surveillance site with a 500-m buffer beyond the site boundary.

### Identification of outcomes

The primary outcomes were T2DM and cardiovascular disease. The secondary endpoints included respiratory and chronic kidney diseases, or cancer.

### Quality control and data management

The surveillance teams, comprising research assistants, laboratory technicians, physicians and coordinators, were trained to follow standardized protocols (Supplementary Table S4, available as Supplementary data at *IJE* online) Their training modules included interviewing techniques, ethics and specific instructions for data variables (demographic, socioeconomic, food security, behavioural risk factors, medication and lifestyle practices, physical measurement and collection of biological samples).

Revalidation of the research teams in study procedures was done at regular intervals during the study to ensure high-quality data collection that was harmonized across surveillance sites. Standardized operating procedures were established for all data collection procedures. Questionnaires were translated into the local languages local to the communities, and back-translated. Equipment used for physical and biological measurements is listed in Supplementary Table S5, available as Supplementary data at *IJE* online, and was regularly calibrated using appropriate controls/standards.

The data management teams reviewed the data collected routinely for completeness and data quality, including using custom computer scripts to assess for biases in data entry, logical inconsistencies, internal correlations, digit preference and measurement drift or bias between machines and observers. Quality control reports were circulated at weekly intervals between the study investigators, to drive continuous evaluation and improvement in study processes. A random subset comprising up to 2% of the study participants, and/or a subset of biological samples, was reassessed to provide additional quality control information. Data collection methods used were ‘field-friendly’, culturally acceptable and minimally invasive in order to reduce participant attrition and improve logistical feasibility.

Personal and clinical data were separated by pseudonymization to enhance data security. All data were encrypted during transmission and stored securely both locally and in a cloud-based infrastructure. Data and all relevant documents will be stored for a minimum of 10 years. Samples collected were split and stored in both South Asia and the UK to ensure long-term (>20 years) sample integrity and preservation. Some laboratory assays on stored samples were done in South Asia and the majority of assays were carried out in the UK or other countries with relevant technologies in the future.

## Data resource use

Data collected in this cross-sectional investigation could be used to assess the epidemiology of T2DM, CVD and other chronic diseases in South Asia. The exploration of possible risk factors for T2DM and CVD could provide a scientific basis for evidence-based public health policy making and interventions. Upon request, the rich resources of SAB are available to researchers from all over the world.

Based on the definition of obesity by WHO, the prevalence of obesity (body mass index ≥30 kg/m^2^) is 6.6% in Bangladesh, 19.7% in India, 33.9% in Pakistan and 15.7% in Sri Lanka. Similarly the prevalence of diabetes, defined as a fasting glucose level >126 mg/dL or a physician diagnosis or current antidiabetic medication, is 11.5%, 27.7%, 25.3% and 24.8%, respectively; and the prevalence of hypertension, defined as a systolic blood pressure ≥ 140 mmHg or a diastolic blood pressure ≥ 90 mmHg or a physician diagnosis or current antihypertensive medication, is 26.7%, 36.9%, 44.5%, 35.0% in Bangladesh, India, Pakistan and Sri Lanka, respectively.

## Strengths and weaknesses

SAB is designed to identify the risk factors and their complex interactions underlying the development of T2DM, CVD and other chronic diseases in South Asians. With intensive data collection, SAB provides representative population samples in four South Asian countries. SAB is the first comprehensive biobank of South Asian individuals. Its large sample size, broad geographical reach and wide range of data collected, including biosamples, make SAB a powerful tool for epidemiological and translational research in South Asian populations. The standardized procedures and rigorous quality control of data collection ensure comparability of study results between and within the partner countries. Further, although random sampling approaches were used in selecting participants, we cannot exclude ‘healthy volunteer’ effects, a common phenomenon in epidemiological research. In addition, advanced phenotyping by imaging (e.g. MRI, DXA or ultrasound) was not feasible across the range of sites studied.

## Data resource access

Reports and major results of SAB will be released regularly on the SAB website [https://www.ghru-southasia.org/]. Any enquiries regarding SAB should be directed to Professor John C Chambers [john.chambers@imperial.ac.uk]. Subject to data privacy requirements and the permissions included in the consent form, individual-level data and samples are available for use to approved investigators.

**Key features**

- The South Asia Biobank (SAB) is the first comprehensive biobank of South Asian individuals, established to identify the risk factors and their complex interactions underlying the development of type-2 diabetes mellitus (T2DM), cardiovascular disease (CVD) and other chronic diseases in South Asians.
- SAB is a cross-sectional investigation in Bangladesh, India, Pakistan and Sri Lanka, starting in November 2018 and ending in March 2020.
- A total of 52 853 participants took part in SAB, and demographic, lifestyle, clinical, environmental and phenotypic data and biological samples are available.
- Interested research collaborators could refer to the SAB website [https://www.ghru-southasia.org/] or contact Professor John C Chambers [john.chambers@imperial.ac.uk].

## Supplementary data

Supplementary data are available at *IJE* online.

## Supplementary Material

dyab029_Supplementary_Data
