# Supplementary material for: Data Resource Profile: Understanding the patterns and determinants of health in South Asians—the South Asia Biobank
Source: Int J Epidemiol. 2021 Jun 18;50(3):717–718e. doi: 10.1093/ije/dyab029 (PMC8271208; doi:10.1093/ije/dyab029)
Supplement: dyab029_Supplementary_Data [file dyab029_supplementary_data.docx]

**Table S1. List of surveillance sites in SAB**

| **Country** | **Name** | **Type** |
| --- | --- | --- |
| **Bangladesh** | Chandipur Jahanabad | Rural |
|  | Khorakhai Cc-parbatipur | Rural |
|  | Jogodishpur Cc, Rajendrapur, Rangpur City Corporation | Urban |
|  | Sheroil Colony Urban Dispensary | Urban |
|  | Vatsail Cc, Badalgachi | Rural |
|  | Kesail, Badalgachi, Naogaon | Rural |
|  | Khalishpur Lal Hospital, Ward 10, Khalishpur, Khulna City Corporation, Khulna | Urban |
|  | Urashi, Ward-8, Babrahasla, Narail | Rural |
|  | Char Gobindapur | Urban |
|  | Kaichan, Birunia, Bhaluka | Rural |
|  | Garakul CC, Jagir, Manikganj | Rural |
|  | Ghosta Cc, Putail, Manikganj | Rural |
|  | Mirpur-1 Urban Dispensary1, st Colony, Darus Salam, Mirpur, Dhaka-1216 | Urban |
|  | Dhankunda Cc, Godnail, Narayanganj | Urban |
|  | Chatibahor, Kompaniganj, Sylhet | Rural |
|  | Gopalnagar CC, Khosbashpur Union, Borura, Cumilla | Rural |
| **Sri Lanka** | Kadawatha | Urban |
|  | Batapotha | Urban |
|  | Peliyagoda | Urban |
|  | Weliweriya | Urban |
|  | Wekada | Rural |
|  | Veyangoda | Urban |
|  | Bokalagama | Rural |
|  | Katana | Urban |
|  | Kochchikade | Urban |
|  | Malwana | Rural |
|  | Kirimatiyana | Urban |
|  | Anavilundawa | Rural |
|  | Mundalama | Rural |
|  | Rural Hospital,Nawagattegama, Sri Lanka | Rural |
|  | Sandalankawa District Hospital, Sandalankawa, Srilanka | Rural |
|  | Karaveddy Dh, Arasady Road Karaveddy | Rural |
|  | Chankanai Dh, Chankanai Sri Lanka | Urban |
|  | Dedigamuwa | Urban |
|  | Pinnawala South | Urban |
|  | Kahahena | Urban |
|  | Aththidiya | Urban |
|  | Kirulapona | Urban |
|  | Blue Mendal / Grandpass Colombo | Urban |
|  | Mattegoda / East | Urban |
|  | Rukmalgama / East B | Urban |
|  | Jamburaliya | Rural |
|  | Sedawaththa, Central Dispensary, Colombo | Urban |
|  | Obesekarapura | Urban |
|  | Angulana | Urban |
|  | Atalugama | Urban |
| **India North** | Hauz Rani | Urban |
|  | J.Jcolony, Budhnagar | Urban |
|  | WZ Block Inderpuri | Urban |
|  | Dasghara Village, Near Todapur 110012 | Urban |
|  | Todapur Village 110012 | Urban |
|  | Z-block , Naraina 110028 | Urban |
|  | Harkesh Nagar,okhla | Urban |
|  | Kalkaji | Urban |
|  | Govindpuri Gali No.3 | Urban |
|  | Govindpuri Gali No.4 | Urban |
|  | Harkesh Nagar,okhla | Urban |
|  | Govindpuri Gali No.2 | Urban |
|  | Harkesh Nagar,okhla | Urban |
|  | Harkesh Nagar B-block | Urban |
|  | Harkesh Nagar C-block | Urban |
|  | Harkesh Nagar D-block | Urban |
|  | Harkesh Nagar E-block | Urban |
|  | Harkesh Nagar F- Block | Urban |
|  | Harkesh Nagar I- Block | Urban |
|  | Harkesh Nagar H- Block | Urban |
|  | Govindpuri Gali No.7 | Urban |
|  | Govindpuri Gali No.8 | Urban |
|  | Govindpuri Gali No. 13 | Urban |
|  | Sanjay Colony A Block Sanjay Colony ,okhla | Urban |
|  | Sanjay Colony Y Block Sanjay Colony ,okhla | Urban |
|  | Sanjay Colony E Block Sanjay Colony ,okhla | Urban |
| **India South** | **Royapettah (Ward 96)** | **Urban** |
|  | Icehouse (Ward 88) | Urban |
|  | Saiva Muthuiya Street (Ward 118) | Urban |
|  | Madhavaperumal Koil Street (Ward 144) | Urban |
|  | Gopalapuram (Ward 112) | Urban |
|  | Mandaveli (Ward 148) | Urban |
|  | Adyar (Ward 152) | Urban |
|  | Thiruvanmiyur (Ward 155) | Urban |
|  | Teynampet (Ward 116) | Urban |
|  | Saidapet (Ward 136) | Urban |
|  | Westsaidpet (Ward 132) | Urban |
|  | Chetpet (Ward 72) | Urban |
|  | KK Nagar,chennai (Ward 128) | Urban |
|  | Kodambakkam (Ward 124) | Urban |
|  | Nugambakkam (Ward 108) | Urban |
|  | Choolai (Chennai Ward 100) | Urban |
|  | Vivekanandapurm (Ward 96) | Urban |
|  | Icehouse (Ward 88) | Urban |
|  | Saiva Muthiah Street Royapettah (Ward 118) | Urban |
|  | BAZAR ROAD, MYLAPORE (Ward 04) | Urban |
|  | Seenuvasa Perumal Street, Gopalapuram (Ward 122) | Urban |
|  | Mandaveli (Ward 173) | Urban |
|  | Besant Nagar,urur Kuppam (Ward 152) | Urban |
|  | Kottivakkam (Ward 115) | Urban |
|  | Dr.Giriapa Road, Teynampet (Ward 116) | Urban |
|  | West Mambalam, Vaazhaithoppu (Ward 136) | Urban |
|  | Ekkattuthangal, chennai (Ward 140) | Urban |
|  | Km Garden (Chennai Ward 100) | Urban |
|  | KK Nagar,chennai (Ward 128) | Urban |
|  | Pullapuram, Kilpauk (Ward 72) | Urban |
|  | Nungambakkam Gangaikarai Puram (Ward 108) | Urban |
| **Pakistan** | Mustafabad, Lahore | Rural |
|  | Shah Jamal, Lahore | Urban |
|  | Ichra, Lahore | Urban |
|  | Kot Araian, Lahore | Rural |
|  | Wahdat Colony, Lahore | Urban |
|  | Barki, Lahore | Urban |
|  | Rhc Chung, Lahore | Urban |
|  | Shadman, Lahore | Urban |
|  | Manga Mandi, Lahore | Urban |
|  | Thq Sabazar (indus Hospital), Lahore | Urban |
|  | Ferozwala Thq, Sheikhupura | Urban |
|  | Jallo, Lahore | Urban |
|  | Manawan | Urban |
|  | Samanabad | Urban |
|  | Chappa Village | Rural |

**Table S2. Response rates in different surveillance sites based on enumerated subjects**

| **Surveillance site** | **Enumerated** | **Registered - with enumeration** | **Registered - without enumeration** | **Response rate - excluding non-enumerated** | **Response rate - including non- enumerated** |
| --- | --- | --- | --- | --- | --- |
| Sri Lanka | 23467 | 11195 | 3640 | 47.7% | 63.2% |
| Pakistan | 5440 | 3340 | 2493 | 61.4% | 107.2% |
| Bangladesh | 17830 | 12894 | 1061 | 72.3% | 78.3% |
| North India | 30901 | 5447 | 4022 | 17.6% | 30.6% |
| South India | - | - | 8621 | N/A | N/A |
| **Total** | **77638** | **32876** | **19837** | **42.3%** | **67.9%** |

**Table S3. Biological samples collected for SAB participants**

| **Sample** | **Volume (ml)** | **N** |
| --- | --- | --- |
| Whole blood | 2 | 1 |
| EDTA plasma | 1 | 2 |
| EDTA buffy coat | 1 | 2 |
| Citrate plasma | 1 | 2 |
| Citrate buffy coat | 1 | 2 |
| Serum | 1 | 6 |
| Spot urine | 2 | 3 |
| RNA (Tempus tube) | 10 | 1 |

**Table S4. Typical composition of each surveillance team**

| **Type of staff** | **Number** | **Job responsibility** |
| --- | --- | --- |
| Medical officer | 1 | - Overall responsibility for the team; - Reporting the clinical and laboratory data collected; - Clinical guidance and counselling of participants. |
| Co-ordinator | 1 | - Co-ordinating the team and oversee data collection; - Implementing quality control protocols. |
| Research Assistants / nurses | 5 | - Registration and consent (1 Pax); - Administering participant questionnaires (2 tables → 2 Pax); - Anthropometry and blood pressure measurements (1 Pax); - Assisting Medical officer in clinical review (1 Pax); - Carrying out household listing (afternoon activity, all). |
| Phlebotomist | 1 | - Collection of blood samples and urine samples. |
| Laboratory technicians | 4 | - Analysing blood samples (1 Pax); - Processing biological samples for storage (afternoon, all). |

*Note: Additional staff were available for transportation and security, as required in respective local contexts.*

**Table S5. Equipment used in SAB**

| **Measurement** | **Device** |
| --- | --- |
| Height | SECA213 stadiometer |
| Weight & bioimpedance | OMRON BF511 (clinically validated) |
| Tape measure | SECA201 measuring tape |
| Blood pressure | Omron M3 IT |
| Spirometry | NuvoAir (https://www.nuvoair.com/) |
| 12 lead ECG | GE Mac2000 |
| Retinal photography | Crystalvue Fundus Camera (NFC700) |
| Point of care tests: | JanaCare Aina (glucose and cholesterol) |
